# Tspan18 is a novel regulator of thrombo-inflammation

**DOI:** 10.1007/s00430-020-00678-y

**Published:** 2020-05-23

**Authors:** Rebecca L. Gavin, Chek Ziu Koo, Michael G. Tomlinson

**Affiliations:** School of Biosciences, University of Birmingham, Birmingham, UK

**Keywords:** Orai1, Endothelial cell, Platelet, Store-operated Ca^2+^ entry, Tetraspanin, Thrombo-inflammation, Tspan18

## Abstract

The interplay between thrombosis and inflammation, termed thrombo-inflammation, causes acute organ damage in diseases such as ischaemic stroke and venous thrombosis. We have recently identified tetraspanin Tspan18 as a novel regulator of thrombo-inflammation. The tetraspanins are a family of 33 membrane proteins in humans that regulate the trafficking, clustering, and membrane diffusion of specific partner proteins. Tspan18 partners with the store-operated Ca^2+^ entry channel Orai1 on endothelial cells. Orai1 appears to be expressed in all cells and is critical in health and disease. Orai1 mutations cause human immunodeficiency, resulting in chronic and often lethal infections, while Orai1-knockout mice die at around the time of birth. Orai1 is a promising drug target in autoimmune and inflammatory diseases, and Orai1 inhibitors are in clinical trials. The focus of this review is our work on Tspan18 and Orai1 in Tspan18-knockout mice and Tspan18-knockdown primary human endothelial cells. Orai1 trafficking to the cell surface is partially impaired in the absence of Tspan18, resulting in impaired Ca^2+^ signaling and impaired release of the thrombo-inflammatory mediator von Willebrand factor following endothelial stimulation. As a consequence, Tspan18-knockout mice are protected in ischemia–reperfusion and deep vein thrombosis models. We provide new evidence that Tspan18 is relatively highly expressed in endothelial cells, through the analysis of publicly available single-cell transcriptomic data. We also present new data, showing that Tspan18 is required for normal Ca^2+^ signaling in platelets, but the functional consequences are subtle and restricted to mildly defective platelet aggregation and spreading induced by the platelet collagen receptor GPVI. Finally, we generate structural models of human Tspan18 and Orai1 and hypothesize that Tspan18 regulates Orai1 Ca^2+^ channel function at the cell surface by promoting its clustering.

## Introduction

Thrombo-inflammation is the complex interplay between thrombosis and inflammation that can result in acute organ damage [1–3]. The process is best characterized in mouse models of ischaemic stroke and venous thrombosis. Central to thrombo-inflammation is the release of ultra-large adhesive strings of von Willebrand factor (VWF) from activated endothelial cells, in response to inflammatory mediators such as interleukin-1 (IL-1), IL-6, tumour necrosis factor α, thrombin, or histamine [2]. A proportion of the VWF strings remain attached to the endothelial cell surface and recruit platelets and leukocytes. The platelet GPIb-IX-V receptor complex mediates transient rolling of platelets on VWF, which may then firmly adhere if activated to induce adhesion of their αIIbβ3 integrins. Leukocytes similarly roll on VWF using their PSGL-1 receptor followed by stable adhesion via β2 integrins. Alternatively, leukocytes may be captured indirectly by platelets via interactions with platelet GPIb-IX-V or P-selectin [2]. In addition, monocytes can bind to VWF having first bound platelet-derived GPIb-IX-V-positive extracellular vesicles [4]. Together, platelet and leukocyte recruitment lead to further recruitment of inflammatory cells, platelet aggregation, and blockage of vessels causing organ damage [1–3].

VWF is stored by endothelial cells in cigar-shaped vesicles called Weibel–Palade bodies (WPBs). VWF is constitutively released at a relatively low level by WPB exocytosis, but can be acutely exocytosed in larger quantities via a mechanism involving Ca^2+^ signaling following endothelial cell activation [5]. We have recently discovered that tetraspanin Tspan18 is important for endothelial cell Ca^2+^ signaling, acute VWF release, and thrombo-inflammation, by interacting with the store-operated Ca^2+^ entry channel Orai1 and promoting its trafficking to the cell surface [6]. This review will describe our current knowledge of Tspan18 expression and function, and will speculate on the molecular mechanism by which Tspan18 regulates Orai1.

## The store-operated Ca^2+^ entry channel Orai1

**Fig. 1:**
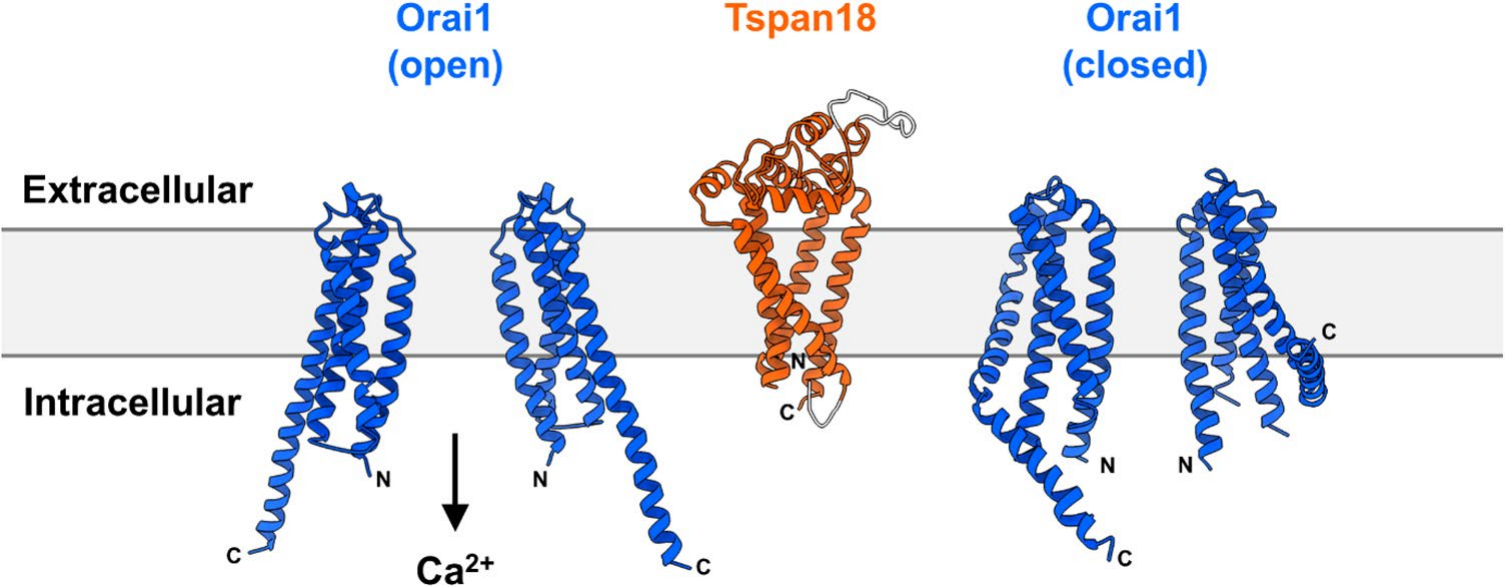
Predicted structures of human Tspan18 and Orai1. Model structures of human Tspan18 (UniProt accession number Q96SJ8) and human Orai1 (UniProt accession number Q96D31) were generated with Phyre2 [62]. Human Tspan18 was modelled based on the crystal structure of human CD81 (PDB: 5TCX) [33]. Of the total protein length, 92% was predicted with 100% confidence (orange region) and the rest was generated ab initio (grey region). The crystal structures of *Drosophila* Orai1 subunits in open (PDB: 6BBF) [63] and closed (PDB: 4HKR) [64] conformations were used as templates for human Orai1 and yielded 100% confidence for 50% and 58% of the total protein length, respectively. The core Orai1 structures are shown; low confidence loop regions on the extracellular and intracellular parts of Orai1 were omitted. Orai1 exists as a hexamer, but only two subunits are depicted for clarity

Orai1 was identified as the long-sought-after store-operated Ca^2+^ entry channel through three publications in 2006 [7–9]. Ca^2+^ influx through Orai1 channels on the cell surface is termed store-operated Ca^2+^ entry, because it is induced by emptying of Ca^2+^ stores in the endoplasmic reticulum (ER). Orai1 has four transmembrane regions and intracellular *N*- and *C*-termini (Fig. 1) [10]. Structural studies suggest that Orai1 functions as a hexamer, forming a channel that mediates a characteristic Ca^2+^ release-activated Ca^2+^ current, termed I_CRAC_. The I_CRAC_ is distinguished by high Ca^2+^ selectivity, a very low unitary conductance, with an inwardly-rectifying current-to-voltage ratio and a very positive reverse potential. The other Orai family members, Orai2 and Orai3, are approximately 60% identical to Orai1 by protein sequence. They are less well studied, but may form their own Ca^2+^ channels or combine with Orai1 to form heteromeric channels [10].

Orai1 appears to be expressed in all cells and is critical in health and disease processes. Indeed, Orai1-knockout mice die at around the time of birth, and Orai1 mutations in humans cause immunodeficiency, leading to chronic infections that are often lethal, and various non-immunological symptoms [11]. As previously mentioned, Orai1 channel opening is induced by emptying of ER Ca^2+^ stores [10]. Such emptying is the downstream consequence of signaling by cell surface receptors, including G protein-coupled receptors (GPCRs), receptor tyrosine kinases, and immunoreceptors such as B- and T-cell antigen receptors. Their stimulation activates phospholipase C, generating the second messenger IP_3_, which induces Ca^2+^ release from IP_3_ receptor Ca^2+^ channels on the ER. The release of Ca^2+^ is sensed by ER-localized STIM1 and STIM2 proteins, which undergo conformational change to engage with and activate Orai1 channels on the cell surface in characteristic clusters, or puncta. The influx of Ca^2+^ replenishes ER stores and, importantly, activates a variety of Ca^2+^-responsive signaling proteins to dictate cell function, such as changes in gene expression through activation of NFAT transcription factors [10].

The immunodeficiency observed in the absence of Orai1 has made this protein a promising drug target to treat diseases caused by an over-active immune system, such as autoimmune and inflammatory diseases [12, 13]. Indeed, five inhibitors of store-operated Ca^2+^ entry have recently entered clinical trials for diseases such as acute pancreatitis, asthma, non-Hodgkin’s lymphoma, and the skin disease plaque psoriasis [12, 13]. However, based on the phenotypes of Orai1-deficient humans and mice, there are concerns that inhibition of Orai1 on every cell in the body could lead to toxic side effects, particularly chronic immunosuppression and reduced muscle strength and endurance [13]. Hence, there is a need to more fully understand the mechanisms of tissue-specific Orai1 regulation, to explore whether novel, more selective inhibitory strategies might inhibit its function in disease without severe general toxicity.

## Tetraspanins regulate the trafficking and clustering of their partner proteins

The tetraspanins are a superfamily of membrane proteins with four transmembrane regions that interact with specific partner proteins, and regulate their trafficking to the cell surface and subsequent lateral mobility and clustering [14, 15]. Tetraspanins appear to exist in all multicellular organisms, from plants to fungi to mammals, and there are 33 tetraspanins in humans [16]. Examples of tetraspanin/partner protein complexes are tetraspanin CD151 and laminin-binding integrins such as α3β1 and α6β1 [17], tetraspanin CD81 and the B-cell receptor co-receptor CD19 [18], and Tspan12 and the Wnt/Norrin receptor Frizzled-4 [19]. The clustering of the partner appears to be the prominent role of CD151 and Tspan12 in these examples, whereas trafficking to the cell surface is the major impact of CD81 on CD19. In each case, tetraspanin deletion or mutation in humans and mice leads to phenotypes that resemble the deletion of the partner [17–19], thus providing genetic evidence for the importance of the interaction. In addition, we and others have shown that a subgroup of six tetraspanins, comprising Tspan5, 10, 14, 15, 17, and 33, and collectively known as the TspanC8s, interact with and regulate trafficking from the endoplasmic reticulum of the ‘molecular scissor’ a disintegrin and metalloprotease 10 (ADAM10) [20–23]. ADAM10 cleaves the extracellular regions from target proteins in a process termed ectodomain shedding. ADAM10 substrates number over 100 and include Notch cell fate regulators, amyloid precursor protein, cadherin adhesion molecules, growth factors, and chemokines. We have proposed a ‘six scissor’ hypothesis, which postulates that ADAM10 is not one scissor, but six different scissors with different substrates depending on which of the six regulatory TspanC8s it is associated with [20–22]. Substantial recent evidence supports this hypothesis [24–32].

Finally, tetraspanins have recently emerged as potential drug targets, following the discovery of the remarkable structure of CD81, the first tetraspanin structure to be solved by X-ray crystallography [33]. CD81 is cone-shaped with a cholesterol-binding cavity and is proposed to undergo a dramatic conformational change if cholesterol is removed [33]. This suggests that tetraspanins might function as ‘molecular switches’ to regulate their partners, but this has yet to be proved. If true, it may be possible to inhibit tetraspanin function using antibodies that lock the tetraspanin in a particular conformation. Alternatively, antibodies could inhibit by down-regulating tetraspanins or disrupting interactions with partners.

## Tspan18 regulates Orai1/Ca^2+^ signaling on endothelial cells

**Fig. 2:**
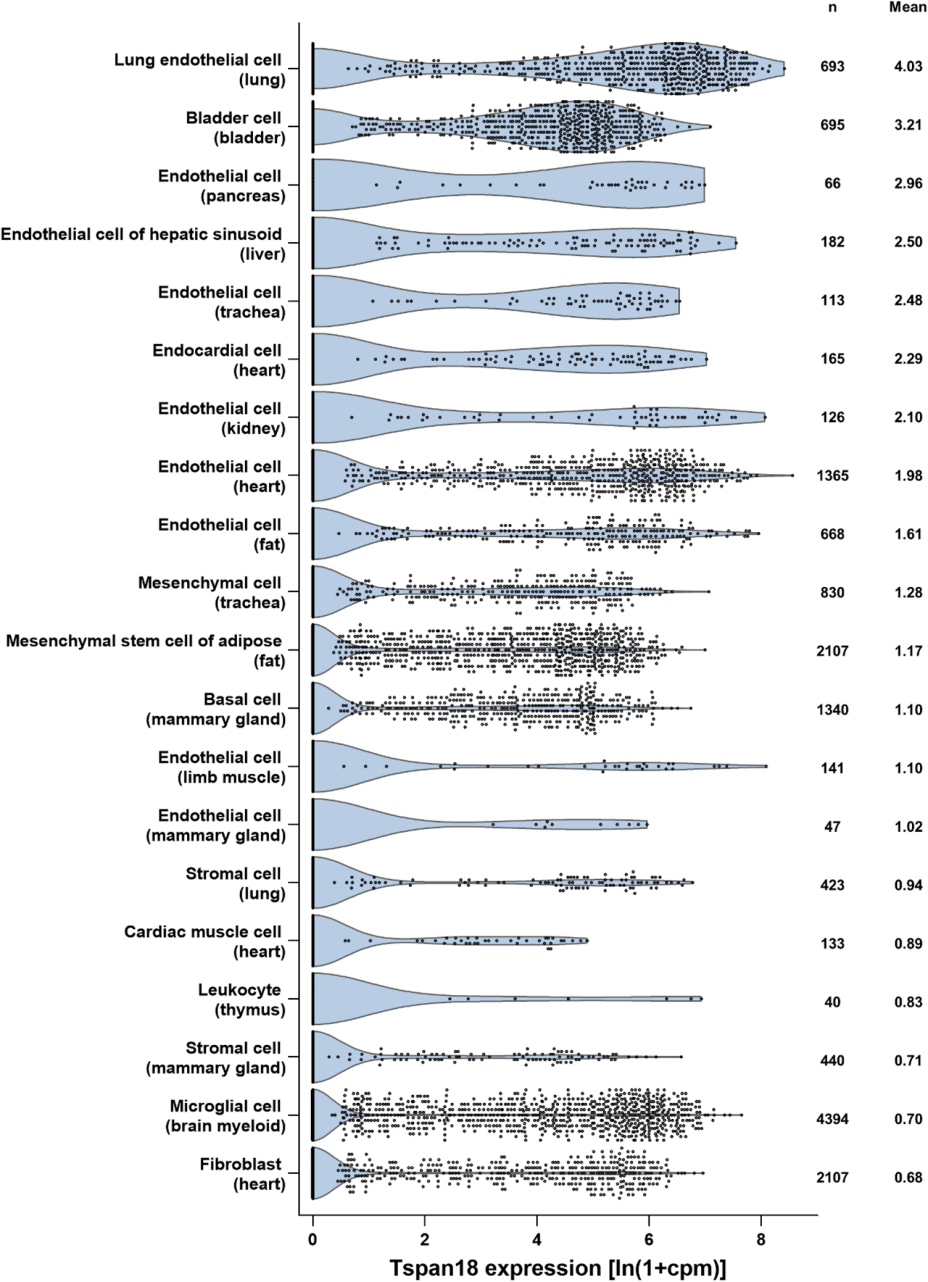
Tspan18 is preferentially expressed by endothelial cells. Mouse Tspan18 expression was analysed from publicly available single-cell RNA-Seq data sets from the Tabula Muris Consortium [34], which contains data from 53,760 cells from 20 organs or tissues of seven mice. Tabula Muris data for gene counts from cells sorted by flow cytometry were normalized to counts per million (cpm) and presented as ln(1 + cpm). Data from the top 20 cell types (tissue of origin in brackets), ranked by mean expression, were visualized as violin plots; n represents number of cells

We recently reported that Orai1 is regulated by Tspan18, a previously uncharacterized tetraspanin in mammalian cells [6]. Our work was initiated by a functional screen for effects of tetraspanins on Ca^2+^ signaling, which found that over-expression of Tspan18, but not other tetraspanins, activates the Ca^2+^-responsive NFAT transcription factor by 20-fold in an Orai1-dependent manner [6]. Furthermore, Tspan18 co-immunoprecipitates with Orai1, but other tetraspanins do not. In the current absence of an effective Tspan18 antibody, we used RT-PCR to demonstrate Tspan18 expression in human endothelial cells, but not most other cell types. This was supported by our analyses of publicly available RNA-Seq data from mouse lung and brain, which showed that Tspan18 is most highly expressed in endothelial cells of these organs [6]. Analysis of single-cell RNA-Seq data from 20 mouse organs provides further support, since 9 of the 14 most highly Tspan18-expressing cell types are endothelial cells (Fig. 2) [34]. Tspan18 is not entirely restricted to endothelial cells, since there is notable expression in bladder cells and weaker expression in certain other cell types (Fig. 2).

We further showed that Tspan18 knockdown in primary human umbilical vein endothelial cells (HUVECs) impairs Ca^2+^ signaling by 55–70% following stimulation with the GPCR agonists histamine or thrombin, similar to Orai1 knockdown [6]. Orai1 surface localisation is 70% reduced following Tspan18 knockdown, suggesting a role for Tspan18 in trafficking Orai1 from the ER. Tspan18-knockout mice are grossly normal and breed as both homozygote knockouts and heterozygotes, the latter yielding offspring at Mendelian ratios. However, the Tspan18-knockout mice display endothelial defects that yield partial protection in two thrombo-inflammatory models: a deep vein thrombosis model (60% reduction in thrombus size) and an ischemia–reperfusion injury heart attack model (50% reduced platelet deposition). Furthermore, there is a 90% and 50% reduction in agonist-induced release of VWF from endothelial cells in vitro and in vivo models, respectively [6]. Together, our data strongly suggest that Tspan18 interacts with Orai1 and regulates its trafficking to the endothelial cell surface, a process which is critical for normal endothelial cell Ca^2+^ signaling and consequent thrombo-inflammatory responses.

In addition to their thrombo-inflammatory phenotypes, Tspan18-knockout mice have a haemostasis defect, as detected by increased blood loss in a tail bleeding assay [6]. Consistent with a role for Tspan18 in endothelial cells, this bleeding phenotype is the result of Tspan18 deletion from non-haematopoietic cells only; this was determined using wild-type/Tspan18-knockout fetal liver chimeric mice [6]. The underlying mechanism remains unclear, since there is no evidence that an acute release of VWF from endothelial cells following injury is critical for haemostasis. However, we hypothesize that in the context of the tail bleeding assay, activation of endothelial cells proximal to the tail cut would lead to Ca^2+^ entry via Orai1 channels, followed by VWF release and platelet recruitment, such that cessation of bleeding would be quicker than in the absence of such VWF release. The haemostasis defect in endothelial-specific VWF-knockout mice [35, 36] would appear to support this hypothesis. However, interpretation of these data is complicated by the strikingly reduced VWF concentration in the plasma of these mice, which is thought to be responsible for increased bleeding [35, 36]. Plasma VWF concentration is normal in Tspan18-knockout mice [6], and thus, we believe that Tspan18/Orai1/Ca^2+^ signaling is important for acute VWF release following cell activation, in contrast to the relatively low-level release of VWF that is known to occur constitutively [5]. We propose that acute VWF release has a role in promoting haemostasis, but this will be challenging to test experimentally.

A recent study identifies the Tspan18 orthologue *tspan18b* as an endothelial-expressed gene in zebrafish, which is required for normal blood vessel development, but its mechanism of action was not investigated [37]. Since VWF is known to promote blood vessel development [38], we propose that impaired Orai1/Ca^2+^ signaling and impaired VWF release may be responsible for the phenotype in *tspan18b*-knockdown zebrafish. Thus, the role of Tspan18 in regulating Orai1/Ca^2+^ signaling in endothelial cells may be conserved across diverse vertebrate species. It remains to be determined whether blood vessel development may also be impaired in Tspan18-knockout mice, although the grossly normal vasculature in adult kidney, pancreas, and ear [6] suggests that any developmental defect may only be subtle or delayed.

An emerging idea in the Orai1 field is that the extent of clustering (promoted by STIM proteins) affects the nature of the ensuing Ca^2+^ signal and downstream cellular responses [39, 40]. We hypothesize that Tspan18 provides an additional mechanism of Orai1 clustering in endothelial cells, and that Tspan18/Orai1 complexes may create a Ca^2+^ channel with membrane dynamics and electrophysiological properties that are distinct from Orai1 alone. A predicted structural model of Tspan18, based on the crystal structure of CD81 [33], is shown alongside the crystal structures of the Orai1 channel in closed and open conformations, to show the comparative dimensions of the proteins (Fig. 1); it has yet to be determined if Tspan18 interacts with higher affinity to the closed or open forms. Mechanistically, Tspan18 may pre-cluster Orai1 channels, resulting in stronger, more rapid Ca^2+^ signaling in response to agonists, which may be critical for endothelial cell function in blood vessel growth and in thrombo-inflammation. Furthermore, the proposed function of tetraspanins as molecular switches [33] might enable Tspan18 to modulate Orai1 via conformational change. We believe that Tspan18 is a potential drug target for the treatment of endothelial-driven inflammatory diseases, cancer angiogenesis, and retinal neovascularisation, which is the most common form of vision loss in the western world. Importantly, targeting Tspan18 may enable Orai1 inhibition in a relatively endothelial-specific manner, so avoiding the likely toxicity that would result from inhibiting this important Ca^2+^ channel on all cells in the body. Targeting tetraspanins is not without precedent, since an antibody to tetraspanin CD37 is in clinical trials for chronic lymphocytic leukaemia [41].

## A subtle role for Tspan18 in platelet Ca^2+^ signaling and aggregation

In addition to the cell types included in Fig. 2, Tspan18 has been detected in human platelets by proteomics [42, 43], and in human and mouse platelets and megakaryocytes at the mRNA level [44–46]. Platelets have a pivotal role in haemostasis, but the bleeding phenotype in the Tspan18-knockout mice is not due to platelet dysfunction, because the experiments with fetal liver chimeras showed that this is a result of Tspan18 deletion in non-haematopoietic cells [6]. Furthermore, Tspan18-knockout platelets aggregate normally in response to the platelet agonists collagen or thrombin [6].

**Fig. 3:**
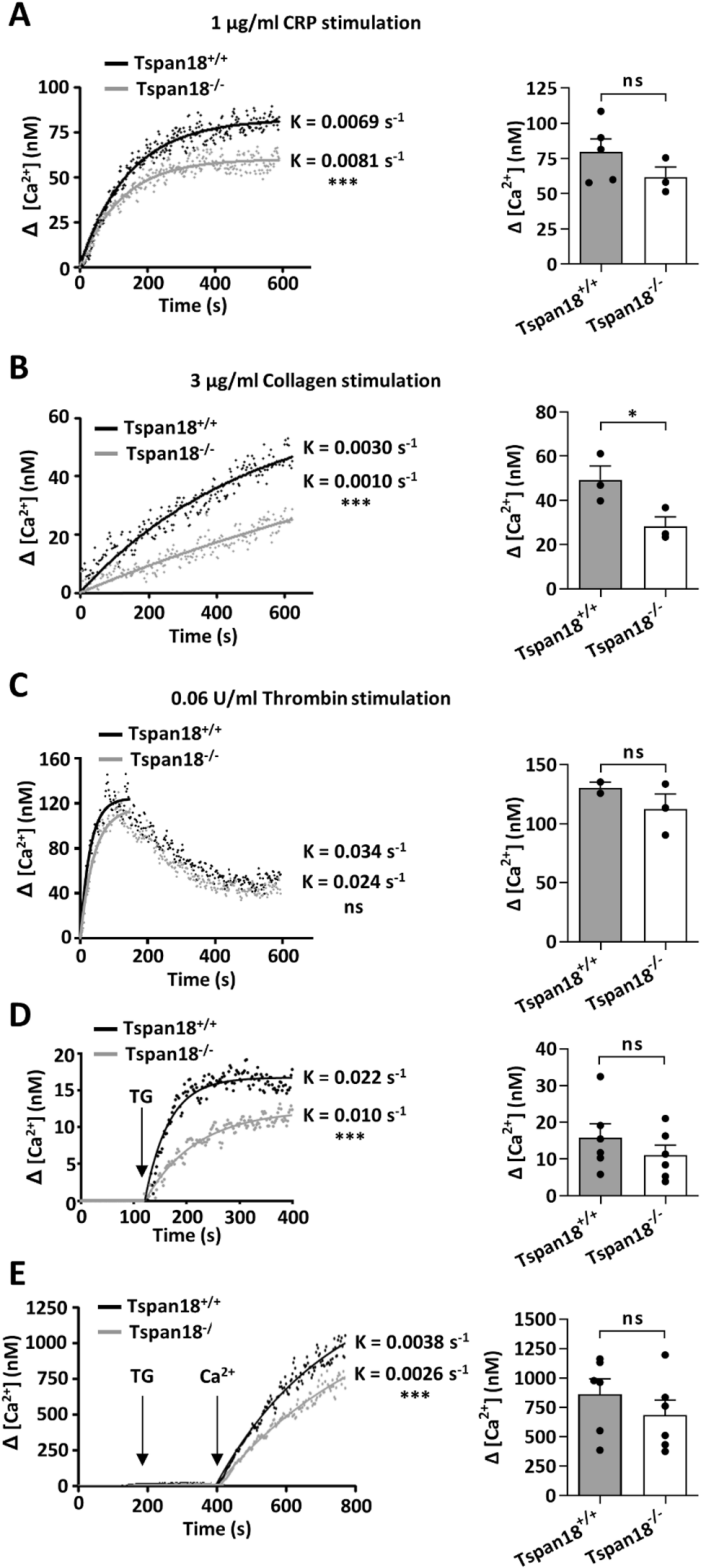
GPVI-induced Ca^2+^ signaling and store-operated Ca^2+^ entry is reduced in Tspan18-knockout platelets. Washed platelets from Tspan18^+/+^ and Tspan18^−/−^ mice were loaded with the Ca^2+^ sensitive dye Fura-2 AM and Ca^2+^ measurements taken using a luminescence spectrophotometer. Collated data were fitted to an exponential one-phase association equation, shown in the left-hand panels, and maximal Ca^2+^ concentration was calculated, shown in the right-hand column. Platelets were stimulated with **a** 1 μg/ml collagen-related peptide (CRP), **b** 3 μg/ml collagen, and **c** 0.06 units/ml thrombin. **d** Platelets were treated in the absence of extracellular Ca^2+^ with thapsigargin (TG) to induce emptying of the intracellular Ca^2+^ stores. **e** The experiment in panel (**d)** was extended by the addition of 1.5 mM Ca^2+^ to the extracellular buffer. The rate constants were compared using an *f* test (*** denotes *P* < 0.001). Maximal Ca^2+^ concentrations were analysed by *t* test (* denotes *P* < 0.05; ns denotes not significant). Error bars represent the standard error of the mean from 2–6 independent experiments. Detailed methods are available online (https://etheses.bham.ac.uk/id/eprint/5903/)

To investigate whether Tspan18 has some role in platelet Ca^2+^ signaling, platelets were loaded with the Ca^2+^-sensitive dye Fura-2-AM. Platelets were stimulated with collagen-related peptide, a specific agonist for the collagen receptor GPVI which activates platelets via non-receptor tyrosine kinase signaling. Intracellular Ca^2+^ concentrations were then monitored with a fluorimeter. The rate of cellular Ca^2+^ accumulation and the maximal Ca^2+^ concentration after 10 min were significantly reduced in Tspan18-knockout platelets (Fig. 3a). A similar result was obtained following stimulation with collagen (Fig. 3b). No significant effect was observed following stimulation with thrombin, which activates platelets via a GPCR (Fig. 3c). To assess store-operated Ca^2+^ entry, platelets were treated in the absence of extracellular Ca^2+^ with thapsigargin, which inhibits sarco/endoplasmic reticulum Ca^2+^-ATPase channels to cause Ca^2+^ store emptying. A reduction in the rate of release and maximal Ca^2+^ increase was observed in Tspan18-knockout platelets (Fig. 3d). Subsequent addition of extracellular Ca^2+^ to the platelets, allowing store-operated Ca^2+^ entry to occur, revealed impaired Ca^2+^ entry in the absence of Tspan18 (Fig. 3e).

**Fig. 4:**
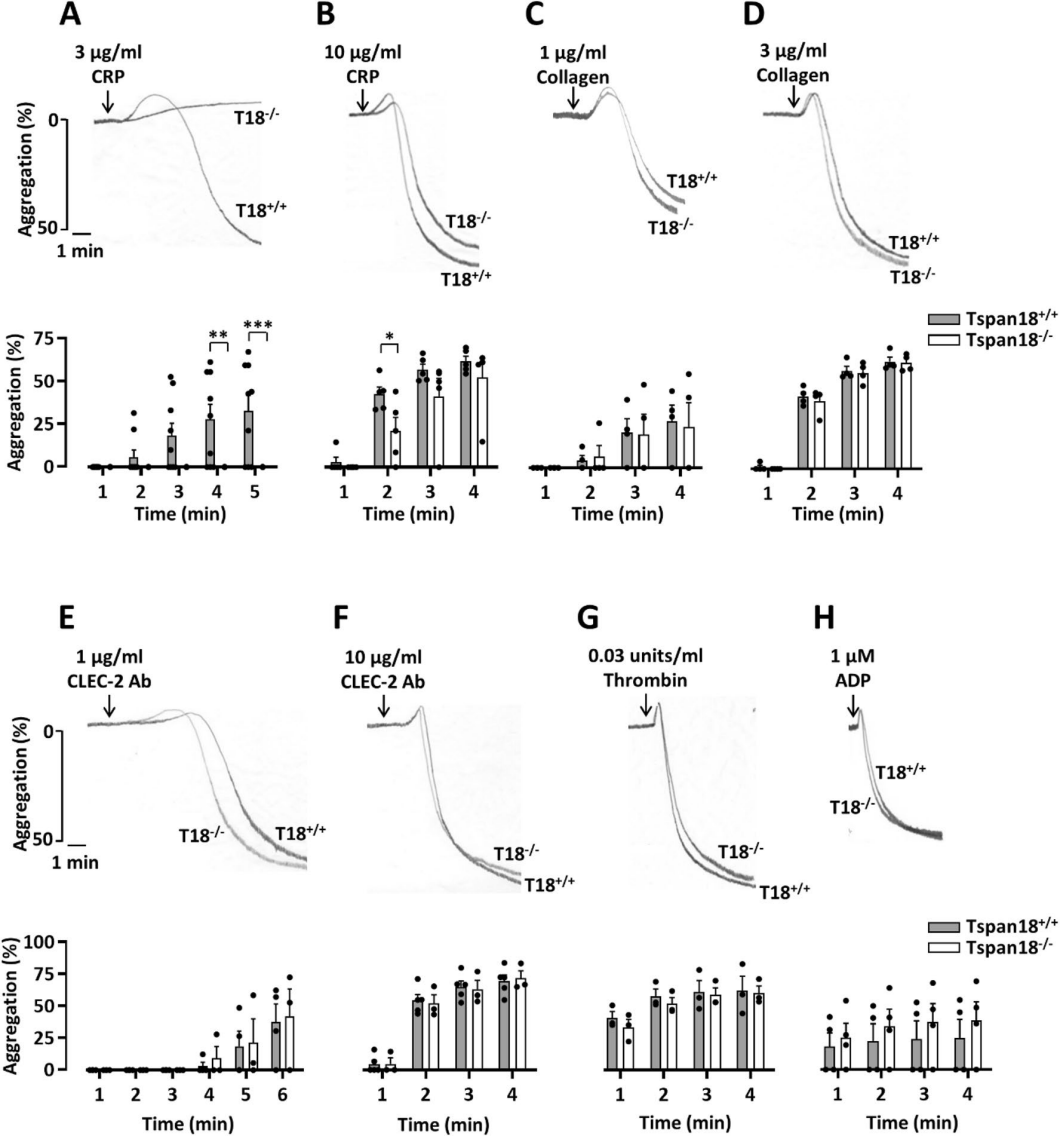
Tspan18-deficient platelets have a specific aggregation defect downstream of GPVI. Mouse platelets were isolated from whole blood taken from Tspan18^+/+^ and Tspan18^−/−^ mice. Washed platelets were activated with **a** 3 µg/ml or **b** 10 µg/ml of the GPVI-specific agonist collagen-related peptide (CRP), **c** 1 µg/ml or **d** 3 µg/ml collagen, **e** 1 µg/ml or **f **10 µg/ml CLEC-2 antibody, or **g** 0.03 units/ml thrombin. **h** Platelets in PRP were activated with 1 µM ADP. Aggregation was measured via light transmission with stirring. Representative traces are shown in the upper panels and quantitated % aggregation per minute shown in the lower panels. A two-way ANOVA was performed on arcsine-transformed data (* denotes *P* < 0.05, ** denotes *P* < 0.01 and *** denotes *P* < 0.001). Error bars represent the standard error of the mean from 3–9 pairs of mice. Detailed methods are available online (https://etheses.bham.ac.uk/id/eprint/5903/)

**Fig. 5:**
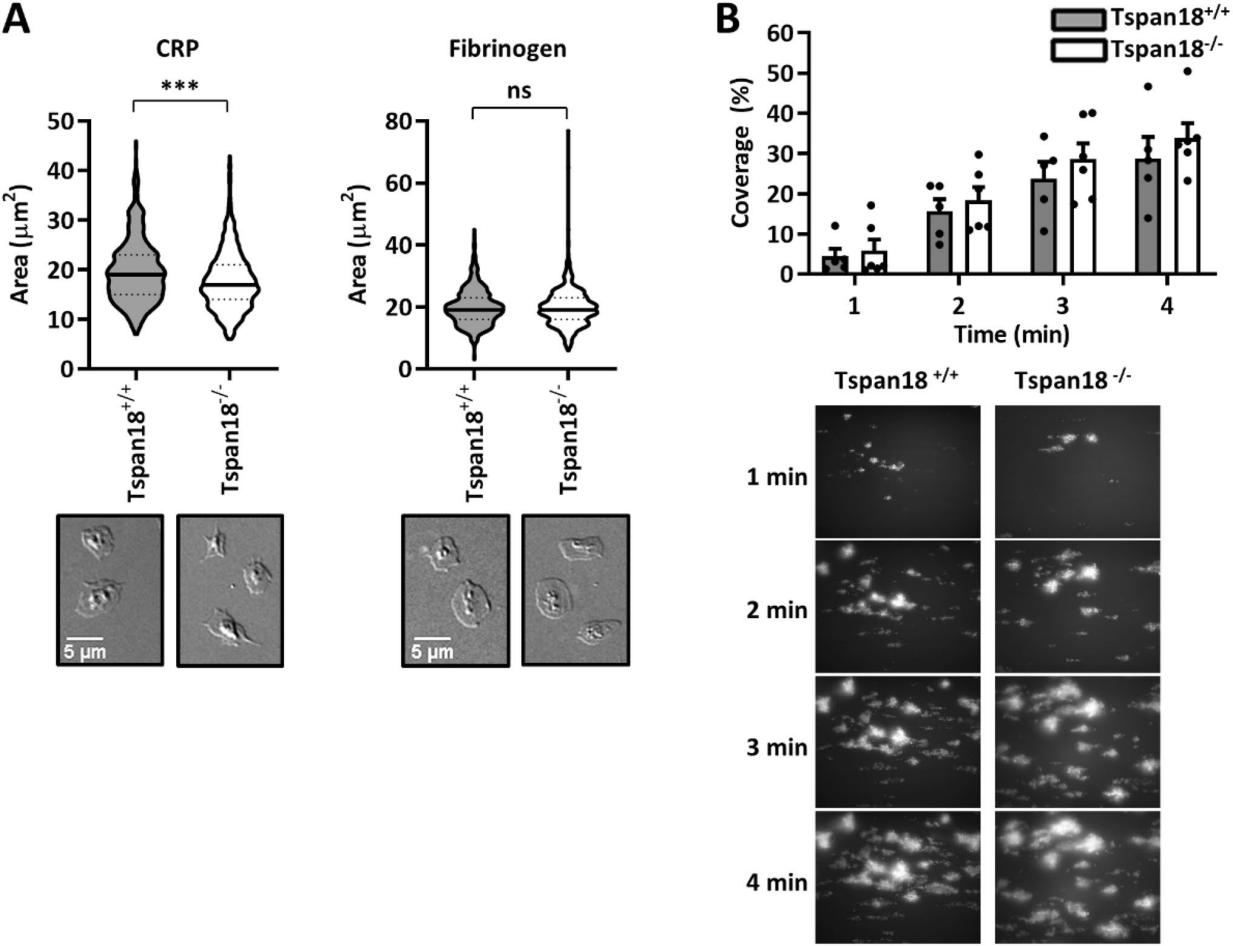
Tspan18-deficient platelets have a specific spreading defect downstream of GPVI, but aggregate formation under flow is normal. **a** Washed platelets from Tspan18^+/+^ and Tspan18^−/−^ mice were exposed to coverslips coated with 10 µg/ml collagen-related peptide (CRP) or 100 µg/ml fibrinogen. The area of the adhered platelets was measured and quantitated. Upper panels show quantitation in the form of violin plots from 4 pairs of mice (150 platelets were analysed per mouse). All data were analysed by *t* test (*** denotes *P* < 0.001, ns denotes not significant). Lower panels are representative DIC microscopy images. **b** Whole blood from Tspan18^+/+^ and Tspan18^−/−^ mice was perfused over a collagen-coated flow cell (30 µg/ml) using the Fluxion Bioflux system at a shear rate of 1000 s^−1^. Coverage of the flow cell by aggregates was measured at 1 min intervals, using thresholding in ImageJ. The upper panel shows quantitated data; a two-way ANOVA with Bonferroni post-test was performed on arcsine-transformed data. Error bars represent the standard error of the mean from three pairs of mice. Lower panels show representative fluorescence images taken at 1 min intervals. Detailed methods are available online (https://etheses.bham.ac.uk/id/eprint/5903/)

To further explore a potential functional role for Tspan18 in platelets, in vitro aggregation assays were employed. Defective aggregation of Tspan18-knockout platelets was observed in response to the collagen-related peptide at the intermediate concentration (Fig. 4a), which was partially rescued at high concentration (Fig. 4b). However, aggregation induced by the physiological agonist for GPVI, collagen, which also binds α2β1 integrin, was normal for Tspan18-knockout platelets (Fig. 4c, d), as we previously reported [6]. Aggregation was also normal when induced by threshold levels of other platelet agonists, such as anti-CLEC-2 antibody, thrombin, or ADP (Fig. 4e–h). To determine whether this GPVI-specific aggregation defect extends to platelet spreading, platelets were plated on glass coverslips coated with collagen-related peptide or fibrinogen; the latter binds to αIIbβ3, the major platelet integrin. Tspan18-knockout platelet spreading was impaired on collagen-related peptide but normal on fibrinogen (Fig. 5a). When aggregate formation on collagen was assessed in whole blood using a flow-adhesion system, no defect in dynamics or total coverage area of platelet aggregates was observed for Tspan18-knockout platelets (Fig. 5b). Together, these data suggest a relatively mild but specific defect in GPVI-induced activation in the absence of Tspan18, which can be overcome at high doses of collagen-related peptide or by collagen, the latter which engages both GPVI and α2β1 integrin; it is possible that signaling induced by the collagen-binding integrin α2β1 [47, 48] is sufficient to overcome the mild GPVI-specific aggregation defect.

**Fig. 6:**
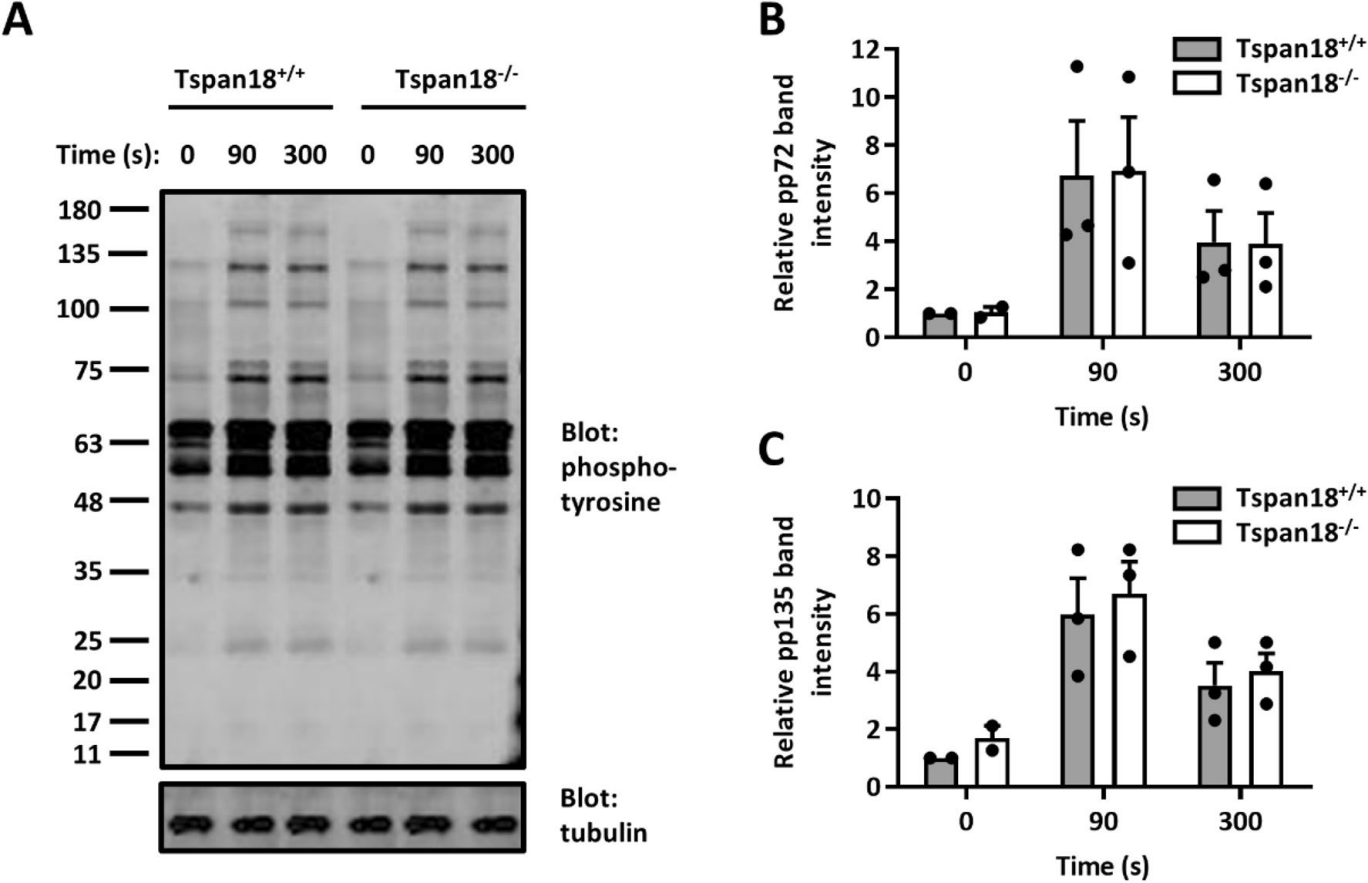
GPVI-induced protein tyrosine phosphorylation is normal in Tspan18-deficient platelets. Washed platelets from Tspan18^+/+^ and Tspan18^−/−^ mice were activated with 3 μg/ml collagen-related peptide (CRP) and samples were taken at 0, 90, and 300 s time points. **a** Protein lysates were separated by SDS-PAGE and blotted with a 4G10 anti-phosphotyrosine antibody. The blots were visualized using the Odyssey Infra-Red Imaging System (LI-COR) and band intensity of **b** the approximately 72 kD band, and **c** the approximately 135 kD band were quantitated. All data were normalized by logarithmic transformation and analysed by two-way ANOVA with Bonferroni post-test. Error bars represent standard error of the mean from three pairs of mice. Detailed methods are available online (https://etheses.bham.ac.uk/id/eprint/5903/)

GPVI signals via an immunoreceptor tyrosine-based activation motif (ITAM) within its associated FcRγ chain, to activate non-receptor tyrosine kinases of the Src, Syk, and Btk families and downstream phospholipase C (PLC) γ2 and Ca^2+^ signaling. The idea that GPVI signaling is impaired in Tspan18-knockout platelets because of impaired Orai1 function is supported by normal upstream signaling, namely tyrosine phosphorylation in response to collagen-related peptide (Fig. 6). Interestingly, in Orai1-knockout platelets, store-operated Ca^2+^ entry is reduced by 80–90%, but the aggregation defect is specific to GPVI [49]. It remains unclear why GPVI-induced platelet aggregation is so reliant on Orai1, but the similarities in phenotype between Orai1 and Tspan18 deficiencies are consistent with the idea that Orai1 is Tspan18-regulated.

## Additional roles for Tspan18: chick embryonic development and links to schizophrenia

Before our recent characterization of Tspan18, the only functional studies on Tspan18 were in chick embryos, in which Tspan18 is required for neural crest cell migration, albeit without a major effect on embryonic development [50, 51]. Neural crest cells are a population of cells that arise transiently during vertebrate development from the ectoderm germ layer. They undergo epithelial-to-mesenchymal transition to migrate and differentiate into a diverse range of cell types. Tspan18 mRNA is expressed in pre-migratory neural crest cells, but is down-regulated when the cells migrate, and forced Tspan18 over-expression prevents migration [51]. There is no evidence that Tspan18 is regulating Orai1/Ca^2+^ signaling in this process. Instead, Tspan18 expression stabilizes the expression of the cell–cell adhesion molecule cadherin 6 at the protein level; down-regulation of cadherin 6-mediated cell–cell interactions is important to allow the cells to disengage from each other and migrate [51]. It has yet to be investigated if Tspan18 regulates cadherin 6 in mammalian cells. Cadherin 6 does not appear to be expressed in endothelial cells in mice [34]. Indeed, cadherin 6 is not expressed by most normal cell types, and is best studied for its expression on renal, ovarian, and thyroid cancers [52]. One cell type in which Tspan18 and cadherin 6 are co-expressed is the platelet. Cadherin 6 blocking strategies suggested a subtle role for cadherin 6 in platelet aggregation via interaction with the major platelet integrin αIIbβ3 [53], but a physical and functional interaction between cadherin 6 and Tspan18 on platelets has yet to be explored.

A genome-wide association study on schizophrenia in the Han Chinese population implicated three single-nucleotide polymorphisms in the 5′ untranslated region of Tspan18 [54]. However, this remains controversial, because these polymorphisms were not replicated in all follow-up studies [55–58]. Nevertheless, altered Tspan18 expression in brain endothelial cell or other brain cell types could affect Orai1/Ca^2+^ signaling and impact brain function and neurological disorders such as schizophrenia. For instance, Tspan18 on endothelial cells could be important for brain function, since endothelial dysfunction in the brain and inflammation is linked to neurological disorders [59, 60]. Alternatively, Tspan18 on microglial cells (Fig. 2) could impact on their capacity to mediate synaptic pruning, an excess of which was recently linked to schizophrenia [61].

## Résumé and outlook

In summary, Tspan18 is important in endothelial cells for the cell surface localization of Orai1, Ca^2+^ signaling, and VWF release. As a result, Tspan18 deficiency is protective in thrombo-inflammatory models of venous thrombosis and ischemia–reperfusion injury in the heart. Ca^2+^ signaling and VWF are both critical to endothelial function in haemostasis, inflammation, blood vessel growth, and angiogenesis [5, 38]. Future work should investigate the importance of Tspan18 in these processes using mouse models of atherosclerosis, ischaemic stroke, and angiogenesis. Generation of Tspan18 monoclonal antibodies will also be important to determine the expression profile of Tspan18 at the protein level. Moreover, such antibodies may have inhibitory activity and, thus, therapeutic potential for thrombo-inflammatory diseases such as deep vein thrombosis, retinal neovascularisation, and cancer angiogenesis to deprive a growing tumour of its blood supply. Finally, it will be interesting to investigate the mechanism by which Tspan18 facilitates Orai1 trafficking to the endothelial cell surface. Once at the cell surface, it will be important to determine whether and how Tspan18 regulates Orai1 clustering and/or channel opening. We may then understand why a process as fundamental as store-operated Ca^2+^ entry is modified by Tspan18 in cells such as endothelial cells, but not in others.
